# Mechanisms of the composite face effect (CFE): Perceptual learning fails to reveal the effect in prototype-based artificial stimuli

**DOI:** 10.1177/03010066261418578

**Published:** 2026-02-03

**Authors:** Ciro Civile

**Affiliations:** 1 3286 University of Exeter, UK

**Keywords:** composite face effect, perceptual learning, face recognition, configural processing

## Abstract

This study investigated the role of perceptual learning in the composite face effect (CFE), which is characterized by reduced accuracy in recognizing the top half of a face when it is combined with the bottom half of another face, particularly when the composite is upright and aligned, compared to when the two halves are laterally offset (misaligned). The misalignment disrupts configural/holistic processing, affecting recognition performance. Experiment 1a (*n* = 96) employed prototype-defined checkerboards to investigate the presence of the composite effect. The advantage of using these stimuli is that expertise can be precisely controlled. Experiment 1b (*n* = 96) aimed to replicate the composite effect using face stimuli, serving as a control and enabling direct comparison of the effect between face and checkerboard stimuli. Both experiments employed a full design that included congruent and incongruent, aligned and misaligned composites to measure the composite effect. Results from Experiment 1a indicated that the composite effect could not be obtained with checkerboard composites, whereas Experiment 1b confirmed the robust presence of the CFE in face stimuli. Based on these findings, we can interpret that perceptual learning does not significantly contribute to the CFE.

## Introduction

Recognition performance for stimuli originating from the same prototype-defined category can significantly improve with prolonged exposure and experience. For example, pre-exposing individuals to a set of checkerboards created by applying random variations to a single prototype enhances their ability to differentiate between similar exemplars. This process, known as perceptual learning, strengthens their capacity to distinguish and remember these stimuli, leading to better recognition accuracy (McLaren & Mackintosh, 2000). Such improvements have been linked to the development of face recognition skills and are thought to underlie some of the most robust phenomena in face perception research, such as the face inversion effect (FIE) (Civile et al., 2014; McLaren, 1997; McLaren & Civile, 2011).

The FIE, where recognizing upside-down faces becomes visibly impaired, was initially thought to reflect the “specificity” of face processing, given its larger magnitude for faces compared to other objects (Valentine & Bruce, 1988; Yin, 1969; Yovel & Kanwisher, 2005). However, further studies challenged this notion by showing that perceptual expertise also plays a crucial role. For instance, it was found that dog breeders exhibited an inversion effect for dog images (Diamond & Carey, 1986), or individuals show an inversion effect for pictures of bodies (Reed et al., 2003), or cars (Rezlescu et al., 2016), houses and words (Albonico et al., 2018). As well, it has been found that an inversion effect can be obtained with sets of artificial stimuli that participants became familiar with only as part of the study familiarization phase for instance, Greebles evoked an advantage at detecting specific parts for upright stimuli but not for inverted ones (Gauthier & Tarr, 1997), or dot patterns (Tanaka & Farah, 1991). Taken all together, these results suggested that familiarity and experience can influence such a robust perceptual phenomenon.

Importantly, several authors have attributed face recognition skills to configural/holistic processing, which relies on subtle differences in the relationships between facial components across the entire face. The FIE has since been used as a robust measure of configural/holistic processing (see Maurer et al., 2002, for a review), and researchers have compared the size of the inversion effect between faces and objects or artificial stimuli to investigate the mechanisms underlying face recognition (e.g., Diamond & Carey, 1986; Gauthier et al., 2004). Therefore, one of the most prominent explanations is that inversion impairs our ability to exploit configural/holistic information, leading to a deficit in recognition performance (Diamond & Carey, 1986). Supporting this, McLaren (1997) demonstrated that perceptual learning mechanisms contribute to the inversion effect using categories of artificial stimuli, checkerboards, that, unlike Greebles, do not have a predefined orientation. Across a series of studies, McLaren showed that participants exhibited an advantage for upright, familiar checkerboards, which was lost when the stimuli were inverted. This advantage depended on two key factors: (1) the checkerboards were drawn from prototype-defined categories (i.e., shared a common configuration of black and white features), as evidenced by the absence of an inversion effect for checkerboards not drawn from prototype-defined categories; and (2) participants had to be familiar with the prototype-defined category of checkerboards, which was demonstrated by the fact that no inversion effect was observed for prototype-defined categories of checkerboards with which participants were not familiar.

Civile et al. (2014) extended McLaren's (1997) work by employing the familiarization task and checkerboard stimuli, but this time incorporating an old/new recognition task, which is commonly used in the FIE literature. Participants first categorized checkerboards into two prototype categories during the familiarization phase (similar to McLaren, 1997), then studied exemplars from these categories as well as from novel categories (the study phase). During this phase, half of the exemplars were presented in their original (upright) orientation, while the other half were rotated 180°, creating upright and inverted conditions. Subsequently, participants completed a recognition task in which all studied exemplars were intermixed with new, unseen checkerboards. They were asked to judge whether they had seen each checkerboard before. Results revealed a clear inversion effect for checkerboards from familiar, prototype-defined categories, with higher accuracy for upright exemplars compared to inverted ones (for a replication, see Civile et al., 2016). Importantly, across a series of experiments, the authors confirmed McLaren's key findings: the checkerboard inversion effect was only observed for stimuli drawn from familiar, prototype-defined categories. No inversion effect was found for checkerboards from non-familiar categories or for familiar checkerboards that were not prototype-defined.

More recently, Civile et al. (2024) and Civile, Quaglia, et al. (2021) further expanded this work by using the same familiarization task and stimuli but employing a matching task designed to ensure robust overall performance with checkerboard stimuli. This approach allows for a direct comparison between the inversion effects for faces and for checkerboards. The findings from these studies reveal that both prototype-defined familiar checkerboards and face stimuli exhibit a comparable inversion effect size and overall performance. Importantly, the results on the checkerboard inversion effect across all these studies have been interpreted within the framework of the McLaren, Kaye, and Mackintosh (MKM) model of perceptual learning (McLaren et al., 1989; McLaren & Mackintosh, 2000), which has recently been supported by simulation work (Civile et al., 2023; Civile et al., 2025). According to this theory, exposure during the familiarization phase to exemplars from a prototype-defined category leads to perceptual learning. Initially, observers focus on the common features of category exemplars, facilitating their association with the category membership. Once these common features are associated with the correct category, their salience diminishes, leaving the unique features in each exemplar more prominent. This feature salience modulation process enables perceptual learning, allowing observers to focus on the unique features of each exemplar, discriminating exemplars from the same category, and recognizing them when upright. Upon inversion, this benefit is lost due to less expertise with inverted stimuli. Overall, these results provide a compelling account of inversion effects for familiarity-based, prototype-defined categories. They also suggest that this mechanism can generalize to other categories, such as faces, supporting the expertise-based explanation of the FIE originally proposed by Diamond and Carey (1986).

Currently, there is clear evidence that perceptual learning is a key mechanism underlying the inversion effect, and the checkerboard studies support this (Civile et al., 2014; Civile et al., 2016; Civile, Quaglia, et al., 2021; Civile et al., 2024; McLaren, 1997; McLaren & Civile, 2011). In contrast, less evidence has been provided to support the robustness of another phenomenon also used as an index of configural/holistic processing, the composite face effect (CFE). This phenomenon refers to the reduced accuracy in recognizing the top half of a face when it is presented in a composite with the bottom half of another face in an upright and aligned position, compared to when the two halves are laterally offset (misaligned). This misalignment disrupts configural and holistic processing. The CFE suggests that, when processing upright faces, internal features are so strongly integrated that it becomes difficult to isolate individual components, causing the entire composite to be perceived as a “new” face (for a review, see Murphy et al., 2017). Importantly, while the inversion manipulation does not directly alter the configural or holistic information, the composite manipulation explicitly does. For this reason, the CFE has become a widely used paradigm in the literature to study the mechanisms of face recognition. Some researchers have employed the CFE to examine differences in configural/holistic processing between upright and inverted faces (Richler et al., 2011). Additionally, studies such as Rezlescu et al. (2017) have provided direct comparisons between the perceptual processes elicited by the inversion effect and those involved in the composite effect.

However, while there is extensive evidence that a robust inversion effect can be obtained with stimuli such as dog or human bodies (Diamond & Carey, 1986; Reed et al., 2003), objects (Albonico et al., 2018; Rezlescu et al., 2016), or artificial stimuli after participants become familiar with them (Civile et al., 2014; Gauthier & Tarr, 1997; Tanaka & Farah, 1991), the role of expertise in the basis of the CFE remains a topic of debate. Some authors have proposed the CFE as an index of face specificity, suggesting that when composite faces are aligned and shown upright, the presence of an intact facial arrangement facilitates access to face-specific processing responsible for this effect (Tsao & Livingstone, 2008). Conversely, others argue that the CFE may reflect a processing strategy employed by object experts. Supporting this view, several studies have reported a composite effect for non-face objects, including cars (Bukach et al., 2010), words (Wong et al., 2019), and Chinese characters (Wong et al., 2012). Additionally, a composite effect has been observed for artificially constructed stimuli such as Greebles and Ziggerins after participants were trained to recognize them (Gauthier & Tarr, 2002; Wong et al., 2009), as well as for images of bodies with expressive postures (Willems et al., 2014).

In contrast, other studies have failed to find a composite effect with non-face objects such as dog images (Robbins & McKone, 2007), neutral body images (Soria Bauser et al., 2011), cars (Cassia et al., 2009), Chinese characters (Hsiao & Cottrell, 2009), and Greebles (Gauthier et al., 1998). These mixed findings continue to fuel ongoing debates regarding the influence of stimulus design, the level of expertise, and factors such as emotional valence across studies that either observe or do not observe a composite effect with non-face stimuli.

For example, two previous studies reported a composite effect for lab-trained, non-face artificial stimuli: Gauthier and Tarr (2002) using Greebles, and Wong et al. (2009) using Ziggerins. Both studies employed a full design. However, initially, Gauthier et al. (1998) did not find a composite effect for Greebles when using a partial design. Similarly, Cassia et al. (2009), which adopted a partial design, did not observe a composite effect for cars, whereas Bukach et al. (2010), using a full design, did find a composite effect for cars. Research has highlighted that composite effects calculated with complete and partial designs do not always correlate (Richler & Gauthier, 2014). A key difference between these designs is that the full design includes both congruent and incongruent composites, presented in aligned and misaligned conditions. In the full design, when measuring the composite effect, a crucial component is the so-called congruency effect, referring to the higher performance for congruent face halves compared to incongruent composites, based on the response required for each half. This effect is typically calculated by subtracting the reduced performance on misaligned, incongruent composites from the robust performance on aligned, congruent composites (see Civile, McLaren, et al., 2021, for an example of a full design with faces).

Recently, Waguri et al. (2021) employed the same familiarization task and prototype-defined categories of checkerboards used in the inversion effect literature (Civile et al., 2014; Civile et al., 2016; Civile, Quaglia, et al., 2021; Civile et al., 2024; McLaren, 1997; McLaren & Civile, 2011) and found that a significant congruency effect could be obtained for checkerboards, demonstrating higher performance for congruent versus incongruent composite stimuli. However, their design included only aligned composites and did not incorporate misaligned composites, thus not capturing the full composite effect as characterized in the literature (Civile, McLaren, et al., 2021; Gauthier & Tarr, 2002; Wong et al., 2009).

This brings us to the key aim of the current study: to determine whether a composite effect can be observed with artificial stimuli such as checkerboards, stimuli previously used to investigate perceptual learning and configural/holistic processing in the inversion effect. To this end, we adopt the same familiarization task and checkerboard stimuli from prior work on the inversion effect, and employ a matching task along with a complete design, typically used in face studies to assess the full composite effect. Our goal is to test whether it is possible to elicit a classic composite effect with checkerboard stimuli (Experiment 1a), similar to that observed with face stimuli (Experiment 1b). This is the first study in the literature to explicitly examine the composite effect for checkerboard stimuli.

## Method

### Participants

**Experiment 1a.** A total of 96 naïve participants (mean age = 25.39, age range = 18–40) were recruited via Prolific (https://www.prolific.com). Participants had an approval rating of at least 90% from previous studies and received monetary compensation in accordance with Prolific Academic's fair pay policies. The sample size was determined through an a priori power analysis conducted using G*Power (Faul et al., 2007). The analysis targeted a medium effect size (*f* = .2) for a one-group, three-measurements design, aiming for a statistical power greater than 0.80 (β = .85). This calculation indicated a total sample of 95 participants, which was increased to 96 to accommodate stimulus counterbalancing across eight groups. This sample size aligns with recent studies that used the same stimuli and similar behavioral designs (e.g., Waguri et al., 2021).

**Experiment 1b.** Similarly, 96 naïve participants (mean age = 23.8, age range = 18–38) were recruited via Prolific, with identical inclusion criteria and compensation as in Experiment 1a. The two experiments were conducted concurrently.

### Materials

**Experiment 1a** utilized the same four prototype-defined categories of checkerboards established in the perceptual learning and inversion effect literature, counterbalanced across participant groups (e.g., Civile et al., 2014). Category prototypes (16 × 16 squares) were randomly generated, with the constraint that each shared 50% of its squares with every other prototype (i.e., 50% black squares and 50% white squares). Exemplars were created from these prototypes by randomly modifying 48 squares—on average, approximately 24 squares would change from black to white or from white to black.

Composite checkerboards were presented at a resolution of 256 × 256 pixels on a gray background. Each composite consisted of a top and bottom half (each containing 16 × 16 squares) of two checkerboard exemplars drawn from the same category. Forty-four of these composites were aligned, while the remaining 44 were converted into misaligned checkerboards by shifting the top half to the left (resulting in a total of 128 composite checkerboards).

**Experiment 1b** used a total of 256 face images (174 × 225 pixels), all standardized to grayscale on a black background. The original images were sourced from the Psychological Image Collection at Stirling open database (https://pics.stir.ac.uk). All images were cropped into a standardized oval shape to remove distracting features such as the hairline and were adjusted to standardize luminance levels (Civile, Quaglia, et al., 2021). These face images were then used to create composite faces following the procedures outlined in Civile, McLaren, et al. (2021). Both experiments were programmed and conducted on the online platform Gorilla.

### The Behavioral Task

**Experiment 1a**. The categorization phase began after participants provided informed consent and received instructions. Participants were presented with exemplar checkerboards from two categories, for example, A and C (64 exemplars from each category; 128 in total). Each exemplar was displayed individually for 4 s in random order, with a fixation cross appearing for 1 s before each stimulus, centered on the screen. Participants were instructed to classify these exemplars into the two categories through trial-and-error by pressing one of two indicated keys on the keyboard (counterbalanced across participants). They received immediate feedback indicating whether their response was correct or incorrect. If a participant did not respond within 4 s, the trial resulted in a timeout.

**Training Phase.** The purpose of this task was for the participants to associate the response keys “x” and “.” with the words SAME and DIFFERENT. They were instructed to press “x” or “.” as quickly as possible when classifying them as SAME or DIFFERENT (counterbalanced). 48 trials (24 SAME, 24 DIFFERENT) were presented randomly one at a time for <1 s after a fixation cross (1 s). They received feedback on each response as correct or incorrect.

**Composite Checkerboard Matching-Task**. This phase involved a matching task following the full design procedure used to measure the composite effect in Civile, McLaren, et al. (2021). However, this time it employed composite checkerboards (128 trials) instead of composite faces. Overall, participants completed 32 trials of “same” aligned, 32 “different” aligned, 32 “same” misaligned, and 32 “different” misaligned composites, split across eight stimulus conditions (each with 16 aligned and 16 misaligned trials): familiar and novel congruent aligned/misaligned, and familiar and novel incongruent aligned/misaligned.

Each trial began with a fixation cross (1 s), followed by a TARGET composite checkerboard stimulus (1 s), an interstimulus interval (1.5 s), and a TEST composite checkerboard stimulus (up to 2 s). Participants were instructed to press either the “x” key or the “.” key (as in the previous training phase) to indicate whether the top halves of the TARGET and TEST stimuli were the same or different. In line with Civile, McLaren, et al. (2021) and Waguri et al. (2021), congruent and incongruent trials were presented in a counterbalanced manner across participants, with aligned and misaligned stimuli randomly intermixed.

**Figure 1.**
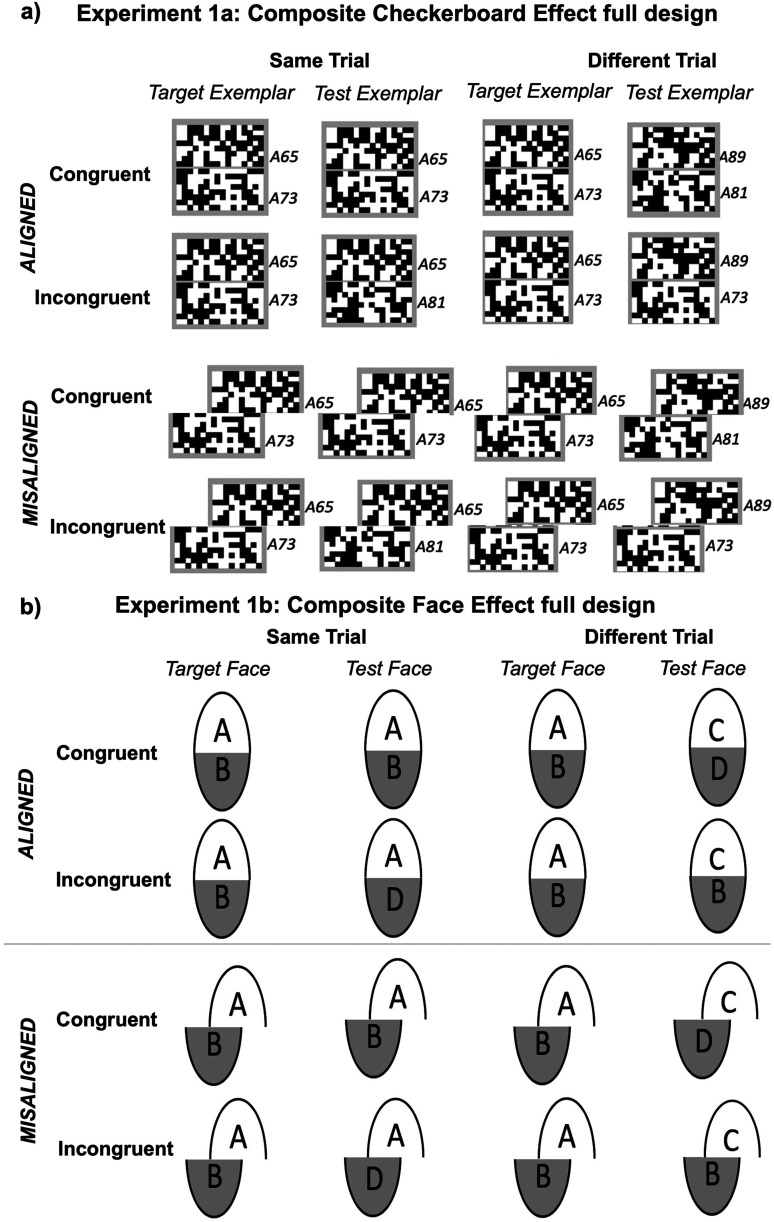
Panel a illustrates the full design for Experiment 1a with aligned and misaligned checkerboard composites. The same design was used for “familiar” and “novel” trials. Panel b illustrates the full design for Experiment 1b, which followed the same logic, except with composite faces instead of checkerboards (Civile, McLaren, et al., 2021).

In the congruent familiar trials, participants first saw a TARGET composite created by combining the top and bottom halves of two exemplars selected from the familiar categories seen during the categorization task (e.g., the top half of exemplar A65 and the bottom half of A73, or the top half of C65 and the bottom half of C73). In the subsequent TEST trial, they saw either the “same” composite (top and bottom halves from the same exemplars) or a “different” composite, where the top and bottom halves were from different exemplars within the same category (e.g., top-half of A89 and bottom-half of A81, or top-half of C89 and bottom-half of C81). Overall, 32 composite images from each familiar category were presented (16 “same” and 16 “different”), randomly ordered. An *A*-target composite was paired with an *A*-test composite, and similarly, a *C*-target was paired with a *C*-test. In the incongruent familiar trials, the TARGET and TEST composites were considered “same” if the top halves matched but the bottom halves differed (e.g., TARGET A65/A81; TEST A65/A73. Conversely, they were considered “different” if the top halves were different but the bottom halves matched (e.g., incongruent familiar: TARGET A89/A73; TEST A65/A73). In the misaligned trials, both congruent and incongruent, the top and bottom halves of each composite were shifted horizontally relative to one another so that they overlapped only across half their length (see Figure 1a). Novel trials were created following the same conditions as for the familiar by using novel sets of checkerboards, not previously familiarized during the categorization task.

**Experiment 1b**. To match the procedure of Experiment 1a, in Experiment 1b, participants first completed a categorization phase. During this phase, they were asked to classify a set of faces—presented one at a time in random order—by pressing one of two indicated keys (counterbalanced). One key corresponded to male faces, and the other to female faces. If participants did not respond within 4 s, the trial was timed out. They received immediate feedback indicating whether their response was correct or incorrect. Each stimulus was preceded by a fixation cross displayed at the center of the screen for 1 s. A total of 128 faces were presented—64 male and 64 female.

**Training Phase.** The same as for Experiment 1a.

**Composite Face Matching-Task**. This followed the full design procedure used in Civile, McLaren, et al. (2021). Each trial began with a fixation cue presented at the center of the screen for 1 s, followed by a TARGET face stimulus for 1 s, an interstimulus interval of 1.5 s, and then a TEST face stimulus (up to 2 s). Participants pressed either the “x” key or the “.” key to indicate whether the top half of the test face was “same” or “different” from the top half of the target face. All composite faces were presented upright and divided into four conditions: Congruent Aligned, Incongruent Aligned, Congruent Misaligned, and Incongruent Misaligned. A total of 64 trials were presented; congruent and incongruent trials were counterbalanced across participants, with stimuli presented in a random mix of aligned and misaligned conditions.

In the congruent aligned trials, participants first saw a TARGET face composite created by combining the top and bottom halves of two different faces (e.g., A–B, where A is the top half and B is the bottom half). In the subsequent TEST face trial, they either saw the same TARGET composite or a new face created by combining the top half of one face with the bottom half of another (e.g., C–D). In the incongruent aligned trials, the top halves matched those of the TARGET faces but had different bottom halves (e.g., A–D), or the top halves differed while the bottom halves remained the same (e.g., C–B).

For the misaligned trials (both congruent and incongruent), the top and bottom halves of each composite were horizontally shifted so that they overlapped across only half of their length (see Figure 1b).

## Results

For Experiment 1a, mean accuracy in the categorization/familiarization task was 75% (range 70–90%), significantly above chance (*p* < .001), indicating that participants learned the two checkerboard categories. In Experiment 1b, the categorization task was administered to match the behavioral procedure of Experiment 1a; mean accuracy was 83% (range 78–95%).

Accuracy in the matching task for both experiments served as the primary measure, from which a *d*-prime (*d*′) sensitivity measure was computed (Stanislaw & Todorov, 1999) for “same” and “different” trials. To calculate *d*’, we used participants’ hit rate (*H*)—the proportion of “same” responses on “same” trials—and false alarm rate (*F*)—the proportion of “same” responses on “different” trials. However, *d*’ is not simply *H* minus *F*; instead, it is the difference between the *z*-transformed values of these rates: *d*’ = *z*(*H*) – *z*(*F*). A *d*’ of 0 indicates performance at the chance level. We evaluated performance against chance to demonstrate that stimulus conditions were recognized significantly above chance (*p* < .001 for all conditions in both experiments). Reaction time data were also examined to ensure that no speed-accuracy trade-off effects influenced the results.

**Experiment 1a**. We conducted a three-way repeated-measures ANOVA with the factors *Congruency* (congruent or incongruent), *Familiarity* (familiar or novel), and *Alignment* (aligned or misaligned). The ANOVA revealed a significant main effect of *Congruency*, *F*(1, 95) = 10.08, *p* = .002, η^2^_p_ = .09, with better detection for congruent stimuli (*M* = 1.80, SE = .08) than for incongruent stimuli (*M* = 1.49, SE = .11). No significant main effects of *Familiarity*, *F*(1, 95) = .026, *p* = .87, η^2^_p_ < .01, or *Alignment*, *F*(1, 95) = .06, *p* = .80, η^2^_p_ < .01, were observed.

Importantly, the interaction between *Congruency* and *Alignment* was not significant, *F*(1, 95) = 2.07, *p* = .15, η^2^_p_ = .02, providing no evidence for a significant composite effect for checkerboard exemplars. Additionally, the interactions of *Familiarity* with *Alignment*, *F*(1, 95) = 2.95, *p* = .09, η^2^_p_ = .03, and *Familiarity* with *Congruency*, *F*(1, 95) = .26, *p* = .61, η^2^_p_ < .01, were nonsignificant. The three-way interaction among *Congruency*, *Familiarity*, and *Alignment* was also nonsignificant, *F*(1, 95) = .24, *p* = .62, η^2^_p_ < .01.

**Figure 2.**
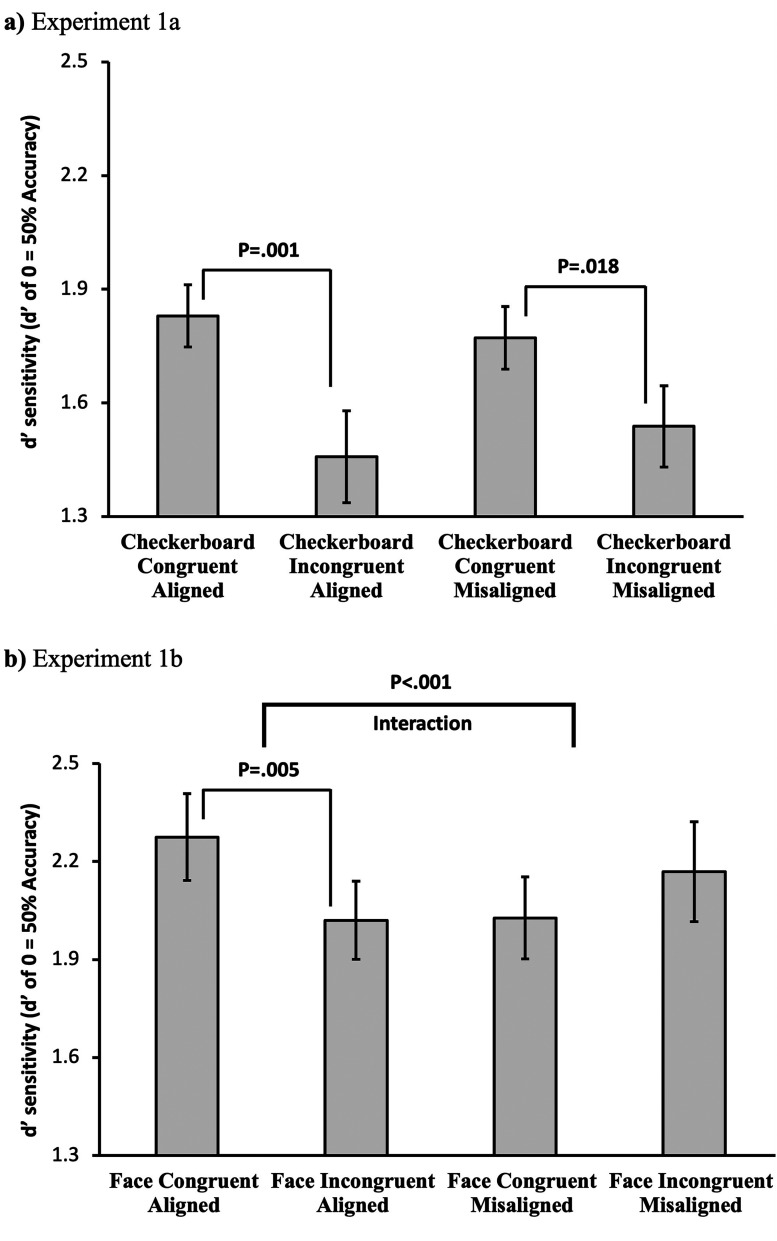
Panel a and Panel b report the results from Experiment 1a and 1b, respectively. In both panels, the *x*-axis shows the stimulus conditions, and the *y*-axis shows *d'*. Error bars represent s.e.m.

To further examine the congruency effect, defined as better performance on congruent versus incongruent trials, we conducted two paired-samples *t*-tests. In aligned trials, participants identified congruent composites (*M* = 1.83, SE = .08) more accurately than incongruent ones (*M* = 1.46, SE = .12), *t*(95) = 3.22, *p* < .001, η^2^_p_ = .09. A similar pattern was observed for misaligned trials, where congruent composites (*M* = 1.77, SE = .08) were better identified than incongruent composites (*M* = 1.53, SE = .10), *t*(95) = 2.40, *p* = .018, η^2^_p_ = .06 (see Figure 2a).

**Experiment 1b.** We conducted a 2 × 2 ANOVA with the within-subject factors *Congruency* (congruent or incongruent) and *Alignment* (aligned or misaligned), which revealed no significant main effects of *Congruency*, *F*(1, 95) = .51, *p* = .475, η^2^_p_ < .01, or *Alignment*, *F*(1, 95) = .92, *p* = .33, η^2^_p_ = .01. Importantly, consistent with previous studies employing the same full design (e.g., Civile, McLaren, et al., 2021), the interaction between *Congruency* and *Alignment* was significant, *F*(1, 95) = 14.62, *p* < .001, η^2^_p_ = .13, indicating a robust CFE.

Further paired-samples *t*-tests revealed a significant congruency effect in aligned trials, with congruent composites (*M* = 2.27, SE = .13) being better identified than incongruent ones (*M* = 2.02, SE = .11), *t*(95) = 2.84, *p* = .005, η^2^_p_ = .08. Conversely, for misaligned trials, the congruency effect was numerically reversed, with congruent composites (*M* = 2.03, SE = .12) being less accurately identified than incongruent ones (*M* = 2.16, SE = .15), *t*(95) = 1.44, *p* = .15, η^2^_p_ = .02 (see Figure 2b).

**Analysis Between the Experiments.** We conducted an additional analysis to directly test whether the composite face effect observed in Experiment 1b was significantly larger than the non-significant effect in Experiment 1a. To do this, we calculated a composite effect index for each experiment by subtracting the congruency effect in misaligned trials from that in aligned trials. Subsequently, an independent *t*-test revealed a significant difference between the two experiments, *t*(190) = 2.08, *p* = .038, η^2^_p_ = .04, indicating a larger composite effect in Experiment 1b, face stimuli (*M* = .40, SE = .10) compared to Experiment 1a, checkerboard stimuli (*M* = .13, SE = .09).

## Discussion

There is strong evidence that perceptual learning is a key mechanism underlying the inversion effect, with checkerboard studies supporting this view (Civile et al., 2014; Civile et al., 2016; Civile, Quaglia, et al., 2021; Civile et al., 2024; McLaren, 1997; McLaren & Civile, 2011). In contrast, evidence for the robustness of another index of configural/holistic processing, the CFE, is more limited. Although a robust inversion effect has been demonstrated with non-face stimuli (e.g., bodies, objects) and with artificial stimuli following familiarization (Albonico et al., 2018; Civile et al., 2014; Gauthier & Tarr, 1997; Rezlescu et al., 2016; Tanaka & Farah, 1991), the role of expertise in producing the CFE remains debated. The present study, therefore, aimed to determine whether a composite effect can be observed with artificial stimuli, specifically, checkerboards previously used to investigate perceptual learning and configural/holistic processing in the inversion effect. We adopted the same familiarization task and checkerboard stimuli used in prior work (e.g., Civile et al., 2014) and employed a matching task with a complete design, as typically used in face studies to assess the full composite effect. Our goal was to test whether a classic composite effect could be elicited with checkerboard stimuli (Experiment 1a), analogous to that observed with faces (Experiment 1b).

The main finding from Experiment 1a was that we did not observe a significant composite effect with checkerboard composites. Consistent with Waguri et al. (2021), we found a significant congruency effect for aligned checkerboard composites but did not observe a significant reduction of this effect in misaligned composites.

The results from the categorization task showed that participants were able to accurately identify the prototypical features shared across the exemplars presented, which determined the specific category membership. Furthermore, a congruency effect was found, which suggest how participants when asked to decide about the top half of the composite were indeed considering both halves (top and bottom) however found easier in the congruent condition to detect the unique features of the composite compared to the incongruent condition. Importantly, the fact that the misaligning manipulation has not had any effect on the size of the congruency effect indicates that participants did not perceive composites as a “whole” stimulus where the configural/holistic representation of the bottom half influenced that of the top half. This would suggest that participants would seem process the composites in a more featural/analytic way (see Maurer et al., 2002 for a review on configural/holistic vs. featural processing), thus no benefits on performance are recorded when configural/holistic processing is disrupted by the misaligning manipulation.

Importantly, Experiment 1b confirmed the effectiveness of the misaligning manipulation at disrupting configural/holistic processing. Hence, the results demonstrated a robust CFE as typically reported in the literature (e.g., Civile, McLaren, et al., 2021). Specifically, a significant congruency effect was observed for aligned face composites, which was numerically reversed when the stimuli were misaligned. An additional analysis comparing the composite effect index between Experiment 1a and Experiment 1b revealed that the composite effect for the face stimuli was significantly larger than that obtained with checkerboard stimuli.

Overall, these results inform the debate over whether the composite effect reflects general perceptual expertise or is specific to faces. Using the same stimuli and familiarization procedure that produce a robust inversion effect comparable to faces (Civile, Quaglia, et al., 2021; Civile et al., 2024), thus demonstrating shared configural/holistic processing mechanisms, we did not observe a composite effect. This suggests that perceptual learning may not underlie the composite effect. This conclusion is consistent with prior studies that failed to find a composite effect for non-face stimuli (e.g., dogs: Robbins & McKone, 2007; neutral bodies: Soria Bauser et al., 2011; cars: Cassia et al., 2009; and Greebles: Gauthier et al., 1998).

One could argue that our results contradict those of Gauthier and Tarr (2002) and Wong et al. (2009), who reported a composite effect for artificially learned stimuli that participants had never prior exposure to in the lab. However, there are two main differences. First, these studies used stimuli—Greebles and certain Ziggerin categories—that can resemble facial features, potentially eliciting configural/holistic processing similar to faces despite being non-face stimuli. Second, their training paradigms emphasized individuation rather than mere categorization. While both approaches aim to develop expertise, individuation training particularly emphasizes subordinate-level discrimination, which has been shown (Wong et al., 2009) to produce a stronger composite effect than basic-level categorization. This suggests that top-down processes like personification or subordinate-level training influence whether configural/holistic processing, and thus the composite effect, emerges, indicating the involvement of an additional component beyond low-level perceptual processes.

It could also be argued that these results may not align with those of Wong et al. (2009), who found a composite effect for Chinese characters in both novices and experts. However, Hsiao and Cottrell (2009) reported the opposite: non-Chinese readers (novices) exhibited a robust composite effect, while native Chinese readers (experts) did not. Hsiao and Cottrell interpreted the stronger configural/holistic processing in novices as reflecting difficulty decomposing character parts due to a lack of knowledge of the writing system; experts, having greater knowledge, were less affected by this decomposition difficulty and therefore showed no significant holistic effects. Wong et al. (2009) proposed that ceiling effects account for the discrepancy: in Hsiao and Cottrell, experts reached *A*′ = 0.95 while novices reached *A*′ = 0.87; when *A*′ was held below 0.95 for both groups, experts also showed robust holistic processing. Nevertheless, subsequent studies have not resolved this discrepancy, and the debate regarding the composite effect for Chinese characters remains open.

A final consideration concerns the stimuli used in Experiment 1a. One might argue that the checkerboards may not contain the configural/holistic information required to elicit a composite effect. That claim would be plausible if this were the first time these checkerboard categories were used to assess perceptual learning in configural/holistic face processing. However, the same checkerboard categories have repeatedly produced an inversion effect in prior studies. Moreover, previous work (e.g., Civile et al., 2014) directly compared prototype-defined checkerboards (the ones used here) with non-prototype checkerboards and found no inversion effect for the latter. Thus, there is no reason to assume that the checkerboards are robust for measuring configural/holistic processing in the inversion effect but not in the composite effect. Likewise, there is no strong basis to claim that Greebles (Gauthier & Tarr, 1997) or dog images (Diamond & Carey, 1986), which have produced inversion effects, should fail to measure the composite effect; indeed, composite effects for these stimuli remain debated (e.g., Robbins & McKone, 2007). To date, inversion and composite effects have been used to index the same underlying process, one by leaving stimuli unaltered (inversion) and the other by directly manipulating them (composite). Future work should further compare these phenomena using identical stimulus sets to clarify subtle differences among the various configural/holistic processes involved in face recognition.

In conclusion, our results clarify mechanisms underlying face recognition skills. We show that perceptual learning expertise with checkerboards does not produce a composite effect (Experiment 1a), whereas the composite effect is robust for faces (Experiment 1b). This pattern suggests the composite effect may rely more on face-specific mechanisms than the inversion effect; however, other factors can influence configural/holistic processing. For example, Liu et al. (2020) showed that task-relevant features (e.g., judging gender or race) and task difficulty modulate the CFE, with larger effects when participants judged the more challenging half of the face; task-irrelevant features had little influence. Ratcliff et al. (2012) found larger CFEs for stimuli associated with high-status occupations, indicating top-down motives can modulate the effect. Similarly, Civile et al. (2019) demonstrated that framing (e.g., claiming faces depicted individuals with autism) reduced the FIE, and Hills et al. (2019) reported that perceived social observation eliminated the FIE, possibly via anxiety-related performance decrements. Together, these findings indicate that, in addition to perceptual learning and configural/holistic processing, top-down, motivational, and arousal/social factors can modulate face processing effects. Future research should dissociate these components.
